# Quality of Diabetes Care Among Older Adults in Germany—Results of the Study on Health of Older People 2022/2023 Compared With Previous National Health Surveys

**DOI:** 10.1111/dom.71184

**Published:** 2026-08-11

**Authors:** Yong Du, Giselle Sarganas, Beate Gaertner, Maike Buchmann, Judith Fuchs, Hannelore Neuhauser, Christin Heidemann

**Affiliations:** ^1^ Department of Epidemiology and Health Monitoring Robert Koch Institute Berlin Germany; ^2^ DZHK (German Centre for Cardiovascular Research), Partner Site Berlin Berlin Germany

**Keywords:** diabetes care, Germany, medication, older adults, self‐management

## Abstract

**Aims:**

The study aims (1) to evaluate the quality of diabetes care considering guideline‐recommended medical therapies, cardiometabolic factors, and self‐management of health behaviours among older adults with diabetes in Germany, and (2) to examine time trends.

**Materials and Methods:**

People aged 66 years and older with diabetes were identified from the examination part of the German national Study on Health of Older People (Gesundheit65+) in 2022/2023. Diabetes care indicators included physical exercise, non‐smoking, non‐obesity, blood pressure (BP) control, guideline‐based medication use for statins, ACEI/ARB, GLP‐1RA/SGLT2i, and aspirin. Temporal trends were examined in a subgroup aged 66–79 years using national health survey data from 1997 to 1999 and 2008 to 2010.

**Results:**

Of 292 older adults with diabetes in Gesundheit65+, 90.6% were non‐smokers, 69.6% were non‐obese, 74.2% achieved BP control, and 45.5% engaged in physical exercise. Overall, 73.6% reported use of antidiabetics, with 24.3% treated by mono‐metformin therapy, 16.9% with insulin (alone or in combination), and 32.4% by other glucose‐lowering medication. Guideline‐recommended medication use was 65.7% for statins overall and 88.4% for ACEI/ARB among individuals with hypertension. Among individuals with cardiovascular disease, it was 36.8% for GLP‐1 RA/SGLT2i and 55.4% for aspirin. Trend analysis in 66‐ to 79‐year‐olds showed a decrease in insulin use and improvements regarding physical exercise, non‐obesity, BP control and guideline‐based medications.

**Conclusions:**

In Germany, most older adults with diabetes meet targets of non‐smoking, non‐obesity and BP control. Use of novel glucose‐lowering medications with cardiorenal benefits is low. Despite improvements over time, guideline‐based medication use should be further improved.

## 1 Introduction

Older people are at high risk for developing type 2 diabetes (T2D) and comprise a substantial and growing proportion of the global diabetes population [1]. In Germany, at least one in five adults aged 65 and older has been diagnosed with diabetes [2]—about 97% of them with T2D [3]. Projections suggest that the total number of adults with T2D will rise from 6.1 million in 2022 to 8.2–12.5 million by 2050, with older individuals constituting the majority of those affected [4]. For older adults living with diabetes, ongoing medical care and effective self‐management are often hindered by the concurrent presence of geriatric syndromes like functional impairments, frailty, and cognitive decline, which complicate treatment and elevate the risk of hypoglycaemia and cardiovascular events [5, 6]. Therefore, diabetes care within this age group must strike a careful balance between glycaemic control and patient‐centred goals tailored to individual needs.

Quality of diabetes care at the population level is typically evaluated through process indicators (e.g., glucose self‐monitoring, regular eye and foot examinations), intermediate outcomes (e.g., control of blood glucose, blood pressure (BP), and cholesterol), and hard endpoints (e.g., diabetes‐related complications and comorbidities) [7, 8]. Quality indicators include adherence to clinical guidelines for managing hyperglycaemia, hypertension, and hyperlipidaemia, alongside the successful implementation of tertiary preventive measures.

While traditional glucose‐lowering therapies—including metformin, sulfonylureas, and insulin—remain predominant among older adults [9, 10], the use of innovative agents, such as glucagon‐like peptide‐1 receptor agonists (GLP‐1RA), sodium‐glucose cotransporter 2 inhibitors (SGLT2i), and dipeptidyl peptidase‐4 inhibitors (DPP‐4i) is increasing [11]. Beyond glycaemic control, GLP‐1RA and SGLT2i demonstrate cardiorenal benefits and support weight management [12, 13, 14, 15] and thus are recommended as first‐line or add‐on therapies to metformin, particularly for those with established atherosclerotic cardiovascular disease (ASCVD) or chronic kidney diseases [16, 17, 18]. Furthermore, clinical guidelines recommend a more intensive reduction in low‐density lipoprotein cholesterol (LDL‐C), primarily by using moderate‐ to high‐intensity statins as the initial treatment [16, 17, 18]. Angiotensin‐converting enzyme inhibitors (ACEI) and angiotensin receptor blockers (ARB) serve as first‐line antihypertensive agents in those with hypertension or those aged 55 years with established ASCVD or multiple ASCVD risk factors, due to their additional benefits in reducing the risk of ASCVD, proteinuria and kidney damage [16, 17]. An antithrombotic therapy by low‐dose aspirin is recommended for people with diabetes and established ASCVD [16, 17]. Apart from medications, self‐management of health behaviours is an essential component of comprehensive diabetes care and serves as the basic measure for the therapy of T2D [19, 20, 21]. Besides weight management in patients with overweight or obesity, all patients with diabetes should quit smoking and increase their physical activity, ideally to 150 min of moderate intensity or 75 min of high endurance intensity per week [16, 21].

Previously, we examined the quality of diabetes care using data from national health surveys conducted in 1997–1999 and 2008–2011, revealing both progress over time and persistent gaps [22] as well as gender disparity [23]. However, these studies were conducted decades ago and were limited to individuals younger than 80 years [22, 23]. Guidelines for the management of diabetes have been updated to incorporate new evidence and evolving best practices [16, 18]. Therefore, in an effort to provide real‐world diabetes care data on older adults, who are often underrepresented in epidemiological studies, we examined data from the most recent nationwide health survey from 2022/2023 among adults older than 65 years in Germany to (1) assess the quality of diabetes care, with a focus on the adherence to guideline‐recommended therapies, cardiometabolic factors, and self‐management of health behaviours, and (2) compare these care indicators with those from the earlier surveys to evaluate whether previously identified gaps have been addressed.

## 2 Methods

### 2.1 Study Designs and Study Populations

Since 1997, the Robert Koch Institute (RKI)—Germany's national public health institute—has conducted three nationwide, population‐based health interview and examination surveys among adults: the German National Health Interview and Examination Survey 1998 (GNHIES98, 1997–1999) [24], the German Health Interview and Examination Survey for Adults (DEGS1, 2008–2011) [25], and the Study on Health of Older People (Gesundheit65+, 2021–2023) [26]. All three surveys used a two‐stage random sampling procedure involving nationwide municipalities and local population registers. While GNHIES98 and DEGS1 were conducted among adults aged 18–79 years, Gesundheit65+ was conducted among adults aged 65 years and older with a baseline and three follow‐up waves. At the time of the final follow‐up from June 2022 to April 2023 (1 year after baseline), participants of Gesundheit65+ were visited at home for an examination. All three surveys were conducted according to principles of the declaration of Helsinki and received approval from the relevant data protection officers. In addition, the two more recent surveys received approval from ethics committees. Prior to participation in these surveys, all survey participants or their legal representatives provided informed consent. Further details of all surveys have been published elsewhere [24, 25, 26].

For the main analyses (aim 1), the study population was restricted to participants with diabetes who attended the final follow‐up examination of Gesundheit65+ in 2022/2023. For the comparative analyses over time (aim 2), study populations from the three surveys were restricted to adults aged 66–79 years with diabetes.

### 2.2 Data Collection and Definition of Study Variables

Data collection comprised self‐reported information by questionnaires or interviews, standardized measurements, and a complete medication review assessing the Anatomical Therapeutic Chemical (ATC) of all medications taken in the 7 days prior to the examination.

#### 2.2.1 Diabetes and Indicators of Diabetes Care

Participants were identified as having diabetes if they reported the condition or were using antidiabetic medications (ATC code A10). The pattern of antidiabetic medications was defined according to the national diabetes surveillance in Germany [27]. Five mutually exclusive groups were distinguished: (1) metformin monotherapy (ATC code A10BA02), (2) other non‐insulin medication only (oral agents other than mono‐metformin, and GLP‐1RA injections (A10B)), (3) insulin only (A10A), (4) combination of insulin and non‐insulin medications, and (5) no glucose‐lowering medication.

Medications used to define guideline‐recommended therapy included GLP‐1RA (A10BJ), SGLT2i (A10BK), DPP‐4i (A10BH), statins (C10AA/C10BA/C10BX), ACEI (C09A/C09B), ARB (C09C/C09D), antihypertensives (C02/C03/C07/C08/C09), platelet aggregation inhibitors (PAI) (B01AC), and aspirin (B01AC06). Cardiometabolic factors considered achieving the BP control target according to measured systolic blood pressure (SBP) < 140 mmHg and diastolic blood pressure (DBP) < 90 mmHg [16, 18] and non‐obesity defined as a body mass index (BMI) < 30 kg/m^2^ according to measured body weight and body height. Self‐management of health behaviour included current non‐smoking classified as former or never smoking, engaging in any physical exercise defined by any sports per week in the past 3 months.

#### 2.2.2 Comorbidities of Diabetes

Comorbidities of diabetes included self‐reported hypertension and cardiovascular disease (CVD), defined by coronary heart disease (including myocardial infarction, chronic symptoms secondary to myocardial infarction, angina pectoris) and stroke (including chronic symptoms secondary to a stroke) in the past 12 months.

### 2.3 Statistical Analysis

First, in analyses restricted to participants in Gesundheit65+, sex‐specific differences within subgroups were tested by second‐order Rao‐Scott χ^2^ tests. As sensitivity analyses, we performed Poisson regression models adjusted for age and educational levels [28] to examine whether observed sex differences remain. Proportion of missing values for diabetes care indicators was low overall, ranging from 0.7% for statin use to 3.8% for physical exercise.

Second, for care indicators in Gesundheit65+ that are comparable in terms of data collection and definition with those of GNHIES98 and DEGS1, we examined temporal trends among persons aged 66–79 years due to age limits in the respective surveys [22]. Prevalence estimates were derived from Poisson regression models, adjusted for sex, age and educational level.

Statistical analyses were performed using Stata software (version 17, StataCorp, USA). Survey‐specific weights were computed to correct for deviations of the study participants from the original target population [26]. Significant group differences were considered at *p* < 0.05 based on two‐sided tests.

## 3 Results

A total of 292 adults (women 47.1%, mean age 76.4 years) had diabetes in Gesundheit65+. Of them, 76.9% reported hypertension and 39.2% had CVD (Table 1). Overall, 24.3% had mono‐metformin therapy, 4.0% used insulin only, 12.9% used a combination of insulin and non‐insulin medications, 32.4% used other antidiabetics, and 26.4% reported no intake of antidiabetics. Use of sulfonylureas was 3.5% (95% CI 1.7%–7.2%), accounting for 10.8% of other non‐insulin medications. No combined use of sulfonylureas and insulin was found (data not shown in Table 1). No sex‐specific differences were found in the age distribution and prevalence of known hypertension and CVD, but women were more often in the primary education group and more likely to report no intake of antidiabetics (Table 1).

**TABLE 1:** Characteristics of older adults with diabetes in the follow‐up in 2022/2023 of the Study on Health of Older People in Germany (Gesundheit65+, *N* = 292).

		Total (*N* = 292)			Men (*n* = 182)			Women (*n* = 110)			*p*
		*n*	%	95% CI	*n*	%	95% CI	*n*	%	95% CI	
Sociodemographic factors											
Age group (years)	66–79	108	57.4	49.5–64.9	72	57.3	47.9–66.2	36	57.5	44.7–69.3	0.979
	> = 80	184	42.6	35.1–50.5	110	42.7	33.8–52.1	74	42.5	30.7–55.3	
Educational level	Primary	137	58.1	49.5–66.1	73	46.7	36.5–57.2	64	70.7	58.3–80.7	0.002
	Middle	82	30.5	23.2–38.9	51	35.8	26.5–46.3	31	24.5	15.1–37.2	
	High	67	11.5	8.6–15.2	54	17.5	12.9–23.3	13	4.8	2.5–8.9	
Self‐reported comorbidities of diabetes											
Hypertension	Yes	209	76.9	69.9–82.6	123	73.0	64.1–80.5	86	81.1	69.4–89.0	0.237
	No	65	23.1	17.4–30.1	45	27.0	19.5–35.9	20	18.9	11.0–30.6	
CVD	Yes	104	39.2	31.8–47.1	64	42.5	31.4–54.5	40	35.3	23.3–49.5	0.483
	No	144	60.8	52.9–68.2	94	57.5	45.5–68.6	50	64.7	50.5–76.7	
Antidiabetic medication pattern											
Mono‐metformin		70	24.3	18.3–31.5	49	25.4	19.1–33.0	21	23.0	13.4–36.5	0.015
Other non‐insulin medication		100	32.4	25.4–40.3	69	36.9	28.7–46.0	31	27.3	17.0–40.9	
Insulin only		19	4.0	2.1–7.5	12	5.2	2.3–11.5	7	2.5	1.0–6.4	
Insulin and non‐insulin in combination		34	12.9	9.1–18.1	25	17.1	11.5–24.7	9	8.3	4.0–16.5	
No medication		67	26.4	19.8–34.3	26	15.3	9.1–24.6	41	38.9	26.4–53.0	

*Note: p* values: comparison between men and women within group. Weighted analyses.

Abbreviations: CI, confidence interval; CVD, cardiovascular diseases.

Regarding adherence to the guideline‐based recommendations, 45.5% of individuals engaged in physical exercise, 90.6% were currently not smoking, 69.6% were non‐obese, and 74.2% achieved BP control (Table 2). Statin use was 65.7% overall. Among those with hypertension, 88.4% used ACEI/ARBs. Among those with CVD, the use of ACEI/ARB, GLP‐1RA/SGLT2i and aspirin was 84.1%, 36.8% and 55.4%, respectively. No sex‐specific differences were observed, except that women with CVD were more likely to use aspirin than men with CVD (Table 2). After adjusting for age and educational level, the prevalence estimates for men and women only marginally changed (Table S1).

**TABLE 2:** Proportion of older adults with diabetes achieving guideline‐based recommendations of diabetes care in the follow‐up in 2022/2023 of the Study on Health of Older People in Germany (Gesundheit65+*, N* = 292).

		Total			Men			Women			*p*
		*n*	%	95% CI	*n*	%	95% CI	*n*	%	95% CI	
Self‐management of health behaviour											
Physical exercise	Yes	133	45.5	36.9–54.3	86	41.2	31.3–51.7	47	50.3	36.9–63.7	0.282
	No	148	54.5	45.7–63.1	91	58.8	48.3–68.7	57	49.7	36.3–63.1	
Non‐smoking	Yes	268	90.6	83.3–94.9	164	87.5	77.2–93.5	104	94.2	80.2–98.5	0.276
	No	18	9.4	5.1–16.7	14	12.5	6.5–22.8	4	5.8	1.5–19.8	
Cardiometabolic factors											
Non‐obesity (BMI < 30 kg/m^2^)	Yes	191	69.6	60.5–77.4	125	73.1	62.6–81.5	66	65.7	52.2–77.1	0.313
	No	90	30.4	22.6*–*39.5	49	26.9	18.5*–*37.4	41	34.3	22.9*–*47.8	
Blood pressure control (SBP < 140 & DBP < 90)	Yes	201	74.2	67.9–79.6	137	78.6	69.8–85.3	64	69.3	57.4–79.0	0.201
	No	85	25.8	20.4–32.1	41	21.4	14.6–30.2	44	30.7	21.0–42.6	
Medication use											
Statin use	Yes	177	65.7	56.9–73.5	112	67.3	58.0–75.5	65	63.8	48.8–76.5	0.672
	No	113	34.3	26.5–43.1	69	32.7	24.5–42.0	44	36.2	23.5–51.2	
ACEI/ARB in persons with hypertension	Yes	178	88.4	82.2–92.6	105	90.4	82.9–94.8	73	86.4	74.6–93.2	0.467
	No	29	11.6	7.4–17.8	17	9.6	5.2–17.1	12	13.6	6.8–25.4	
ACEI/ARB in persons with CVD	Yes	86	84.1	74.4–90.6	51	79.3	63.4–89.5	35	91.0	75.6–97.0	0.196
	No	18	15.9	9.4–25.6	13	20.7	10.5–36.6	5	9.0	3.0–24.4	
GLP‐1RA/SGLT2i in persons with CVD	Yes	36	36.8	24.2–51.4	24	38.2	22.8–56.5	12	34.7	18.6–55.2	0.768
	No	68	63.2	48.6–75.8	40	61.8	43.5–67.2	28	65.3	44.8–81.4	
Aspirin in persons with CVD	Yes	53	55.4	43.0–67.1	30	44.1	28.1–61.4	23	71.5	54.5–84.1	0.026
	No	51	44.6	32.9–57.0	34	55.9	38.6–71.9	17	28.5	15.9–45.6	

*Note: p* values: comparison between men and women within group. Weighted analyses.

Abbreviations: ACEI/ARB, angiotensin‐converting enzyme inhibitor/angiotensin receptor blocker; BMI, body mass index; CI, confidence interval; CVD, cardiovascular disease; GLP‐1RA/SGLT2i, glucagon‐like peptide‐1 receptor agonist/sodium‐glucose co‐transporter 2 inhibitor; SBP < 140 & DBP < 90, systolic blood pressure < 140 mmHg & diastolic blood pressure < 90 mmHg.

Independent of guideline recommendations, the overall use of GLP‐1RA/SGLT2i and DPP‐4i was 23.5% and 24.6%, respectively (Table S2). Use of any antihypertensives, antihyperlipidemics and PAI was 88.8%, 67.1% and 38.7%, respectively. ACEI/ARB accounted for 88.0% of all antihypertensives with a prevalence of 76.6%. Aspirin accounted for 91.3% of all PAIs with a prevalence of 35.1%, while statins accounted for 95.5% of all antihyperlipidemic agents.

Regarding the time trend for the antidiabetic medication pattern among adults aged 66–79 years, insulin monotherapy decreased between 2008–2011 and 2022/2023 (Figure 1). Meanwhile, the use of insulin in combination and metformin monotherapy remained largely unchanged, while the use of other non‐insulin medications increased. In the ‘other non‐insulin’ medication group, the proportion of sulfonylurea use decreased from 99.1% in 1997–1999 to 61.7% in 2008–2011, and further to 1.3% in 2022/2023, whereas in the ‘combined insulin & non‐insulin’ medication group, the proportion fell from 90.1% in 1997–1999 to 25.0% in 2008–2011, and further to 0% in 2022/2023 (data not shown in Figure 1).

**FIGURE 1:**
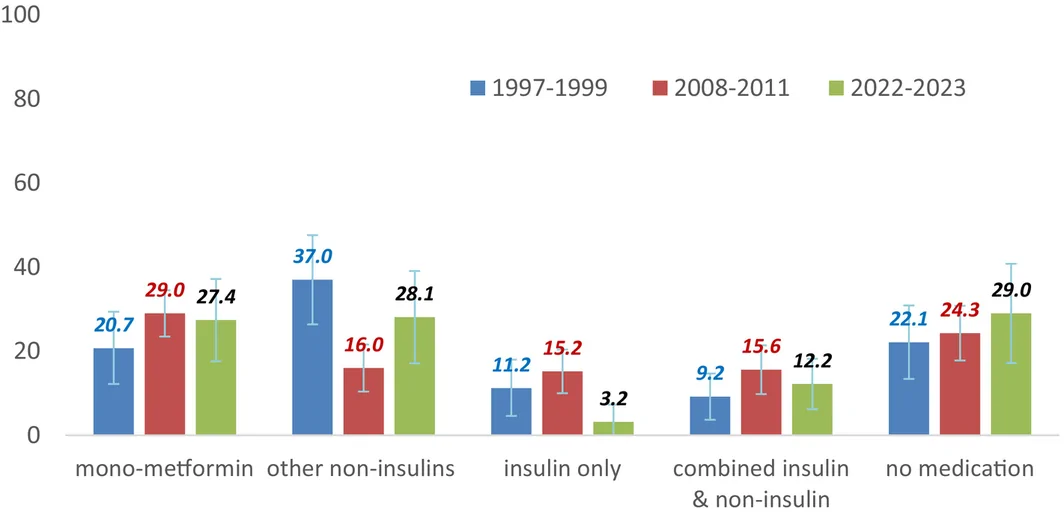
Comparison of antidiabetic medication use pattern among older adults with diabetes aged 66–79 years in the national health surveys conducted in 1997–1999 (GNHIES98, *n* = 151), 2008–2011 (DEGS1, *n* = 328) and 2022/2023 (Gesundheit65+, *n* = 108) in Germany. Weighted analyses, adjusted for age, sex and educational level.

In terms of adherence to guideline‐based recommendations, physical exercise increased from 16.7% in 1997–1999 to 49.3% in 2008–2011, reaching 54.9% in 2022/2023, whereas the proportion of non‐smoking remained at almost 90% (Figure 2). In 2022/2023, 68.5% of individuals were not obese, which is higher than in 2008–2011 but similar to 1997–1999. A higher proportion of adults achieved BP control in 2022/2023 than in 2008–2011 and in 1997–1999. A steady increase over time was observed in statin use from 8.1% in 1997–1999 to 70.1% in 2022/2023 and in ACEI/ARB use among those with hypertension from 53.0% in 1997–1999 to 91.4% in 2022/2023. Among individuals with CVD, use of ACEI/ARB and aspirin increased from 1997–1999 to 2008–2011 but stagnated since then, whereas GLP‐1RA/SGLT‐2i were only available in 2022/2023 (Figure 2).

**FIGURE 2:**
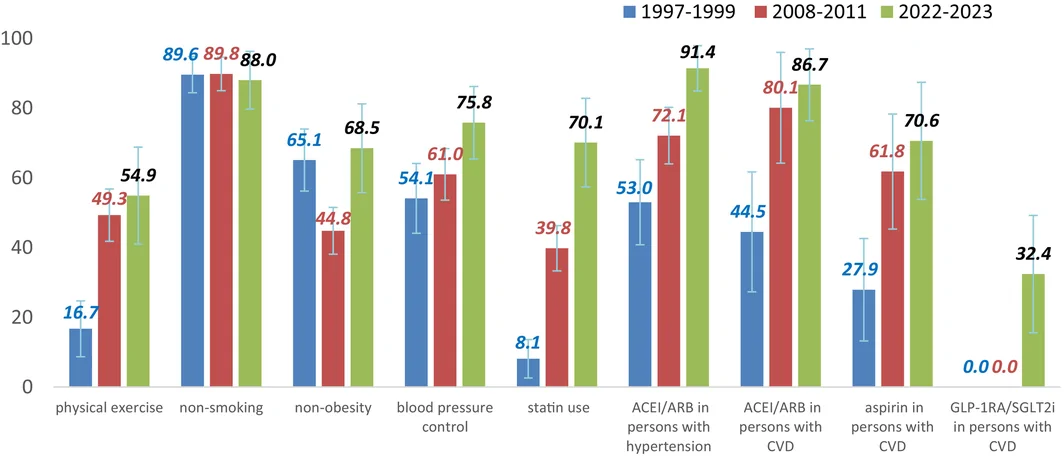
Comparison of diabetes care indicators among older adults with diabetes aged 66–79 years in the national health surveys conducted in 1997–1999 (GNHIES98, *n* = 151), 2008–2011 (DEGS1, *n* = 328) and 2022/2023 (Gesundheit65+, *n* = 108) in Germany. Weighted analyses, adjusted for age, sex and educational level. ACEI/ARB, angiotensin‐converting enzyme inhibitors/angiotensin receptor blocker; CVD, cardiovascular disease; GLP‐1RA, glucagon‐like peptide‐1 receptor agonists; SGLT2i, sodium‐glucose cotransporter 2 inhibitors.

## 4 Discussion

### 4.1 Main Findings

In 2022/2023, almost three‐quarters of older adults with diabetes in Germany took antidiabetic medications, with fewer than one in five using insulin. About 90%, 70% and 75% of individuals achieved the target of non‐smoking, non‐obesity and BP control, respectively, whereas only less than one half engaged in physical exercise. Guideline‐recommended medication use was common for ACEI/ARB, moderate for statins and aspirin, but comparatively low for GLP‐1RAs/SGLT2i. Trend analysis shows a decrease in insulin use and an improvement in physical exercise, non‐obesity, BP control and use of guideline‐based medications, while non‐smoking remained constant at around 90%.

### 4.2 Patterns of Antidiabetic Medication Use

Insulin use in our study (16.9%) was lower than in earlier studies based on GEDA 2021/2022‐Diabetes data (37%) [29]; statutory health insurance (SHI) claim data from 2015/2017 (27.6%–30.0%) [10] and from 2018 (35%) [30], and Diabetes Prospective Follow‐up Registry (DPV) data from 2017 to 2022 (52.4%–56.3%) [31]; but similar to that based on patients enrolled in a disease management program (DMP) for T2D in 2024 (20.0%) [32] and regional SHI claims data from 2022 (20.3%) [33]. Clinical guidelines recommend the reduction of hypoglycaemia‐causing medications such as insulin and sulfonylureas in older adults [34, 35]. Accordingly, an increasing delay in starting insulin therapy was observed based on data from general practices in Germany between 2010/2011 and 2016/2017 [36], suggesting a shift from early insulin treatment toward a more individualized approach. Furthermore, SHI claims data indicate that the total insulin use has been slowly declining since 2017 [37]. The overall low insulin use and its downward trend as well as the sharp decline in sulfonylureas use found in our study may reflect current clinical practice and efforts toward deprescribing in older adults with T2D [38], considering the risk of hypoglycaemia and associated falls [16, 17, 18]. This may also possibly be due to an increased use of new antidiabetics, such as GLP‐1RAs and SGLT2i. A GLP‐1RA is preferred to insulin initiation for most adults with T2D [39], delaying the need for insulin therapy [36].

More than a quarter of older adults with diabetes did not receive any antidiabetic medication. This is consistent with the results for 45‐ to 79‐year‐old participants with T2D in the earlier health surveys DEGS1 and GNHIES98 [22] and for DMP‐registered T2D patients [32], but higher than the results (13%) of adults aged 45 years and older with T2D based on the more recent telephone survey GEDA 2021/2022‐Diabetes [29]. SHI claims data with large sample sizes showed that more than 35% of T2D patients aged 18 years and older did not receive antidiabetic drugs [30, 40]. However, despite differences in survey design and, consequently, in recording of medication, the above‐mentioned previous health surveys all revealed that a large proportion of those without antidiabetic medication report treatment by diet or other lifestyle interventions [22, 29], which are considered as basic non‐medication care for T2D [21].

### 4.3 GLP‐1RA and SGLT2i Use

SHI claims data from 2018 suggested that only 5% and 11% of treated T2D patients received GLP‐1RA and SGLT2i, respectively [30]. More recent data from DMP showed that the use of GLP‐1RA and SGLT2i was 10.9% and 23.5%, respectively [32]. This is consistent with the observed increasing trend of GLP‐1RA and SGLT2i between 2017 and 2022 based on DPV data [31] as well as our finding of GLP‐1RA and/or SGLT2i use overall in 2022/2023. Among T2D patients with CVD, the use of GLP‐1RA/SGLT2i based on SHI claims data was 13.0% in 2020 [40] compared to 36.8% in our study. These findings suggest that there is room for more frequent use of GLP‐1RAs and SGLT2is, given that the German guideline for management of T2D recommends the early use of these drugs in patients with cardiovascular or renal diseases [18], which are common among older adults with diabetes [41]. Yet, concerns about the use of these drugs, such as unintended weight loss and associated sarcopenia [42], particularly in older, frail populations, should be taken into account in clinical practice. Furthermore, a socioeconomic gradient in the use of GLP‐1RA and SGLT2i was observed with the lowest use being found in the most deprived region [43]. Women were less likely to be treated with SGLT2i than men [31, 43]. We also observed a tendency for women to use GLP‐1RA/SGLT2i less frequently than men. As the German healthcare system provides insured individuals, which are around 90% of the population, with access to all required diabetes medications, further investigation is needed to understand disparities in the use of GLP‐1RA and SGLT2i. Ensuring overall and equitable access to evidence‐based pharmacotherapy remains a critical priority for improving care.

### 4.4 Medication Use for CVD Prevention

Hypertension and hyperlipidaemia are both common concomitant conditions of diabetes and major risk factors for ASCVD. It is therefore encouraging that a high proportion of individuals in our study receive antihypertensive and antihyperlipidaemic medications as also found in other studies [10, 30]. Among T2D patients aged 65 years and older identified from a regional SHI claims data, the prevalence of antihypertensive use was over 90% overall, and approximately 83% of men and women received ACEI/ARB by 2015–2017, which had changed little since 2010–2012 [10]. During the same period, the use of lipid‐lowering medication increased, reaching 47.0% for women and 57.9% for men by 2015–2017 [10]. The considerable increase in statin use over time in our study, reaching up to two‐thirds of the older adults with diabetes, aligns with the global diabetes target of at least 60% of people aged 40 years and over with diagnosed diabetes receiving statins [44]. This increase is likely to be largely attributable to successive updates in clinical guidelines that, for example, have progressively lowered target levels for LDL‐C [16, 45].

The benefits of aspirin for CVD primary prevention are controversial and may be outweighed by the risk of major gastrointestinal bleeding [46, 47]. Low‐dose aspirin is thus primarily recommended for CVD secondary prevention [16, 17, 18]. Among DMP‐enrolled T2D patients with ASCVD, use of PAI was 70.4% in 2015 and 67.5% in 2024 [32], which is slightly higher compared to our findings for aspirin use (55.4%) among individuals with CVD.

Overall, the significant increase in the use of ACEI/ARB, statins, and aspirin over time suggests a continuous improvement in secondary prevention of CVD among adults with diabetes. However, according to guideline‐based recommendations, there is room for further improvement regarding the use of statins among adults with diabetes and the use of aspirin among those with diabetes and comorbid CVD.

### 4.5 Cardiometabolic Factors and Self‐Management of Health Behaviours

Three‐quarters of older adults with T2D achieved the target of SBP/DBP < 140/< 90 mmHg in our study, which is comparable to the results in a population‐based study in 2014 (69%), despite the use of a more stringent BP goal of < 140/< 80 mmHg [48]. Of DMP‐enrolled T2D patients, 59.5% achieved the BP target of < 140/< 90 mmHg in 2024 [32]; yet, only T2D patients with known arterial hypertension were included in this DMP indicator [32]. The moderate to high proportion of T2D patients achieving the BP targets is close to the global diabetes target of 80% of people with diagnosed diabetes having a good blood pressure control [44]. The upward trend found here and in other studies [10, 32] is likely due to the widespread use of antihypertensive medications, particularly ACEI/ARB [10].

The prevalence of obesity among adults with T2D in Germany is high, with different sources indicating that more than one in three to half of T2D patients are affected [29, 32, 40, 49]. Obesity in patients with T2D decreases with age, falling from over 60% in those aged under 60 years to 30% in those aged over 80 years [32]. Among T2D patients aged 65 years and older, the prevalence of obesity was reported at 37.6% in a regional population‐based study in 2016 [50], compared to our findings of 30.6% in 2022/2023. A slight increase in obesity was observed between 2004 and 2015 among DMP‐enrolled T2D patients [49], and between 2014 and 2020 among T2D patients derived from SHI claim data [40]. Our findings among 66‐ to 79‐year‐olds with diabetes suggest that the upward trend in obesity may have reversed over the past 10 years, indicating an improvement in weight management. While this needs to be confirmed by studies with a broader age range and larger sample size, the decreased prevalence of obesity over the past decade may be partly attributed to the decreased use of insulin, which is rather known to be associated with weight gain [51], and the increased use of GLP‐1RA and SGLT‐2i, which are known to cause weight loss [12].

People with diabetes are more likely to be physically inactive than the general population [52] despite exercise recommendation of guidelines [16, 19, 20, 21]. A pooled analysis of 13 population‐based health surveys conducted between 1997 and 2018 in Germany found that 47.8% of adults with diabetes aged at least 50 years reported engaging in leisure‐time physical activities every week [52]. This is consistent with the observed 45.5% in the present study. Although physical exercise increased between 1997–1999 and 2008–2011 in the subgroup of 66‐ to 79‐year‐olds, this trend has not continued into the last decade. Furthermore, over half of older patients still do not meet the minimum time threshold [29], underscoring the necessity of promoting structured, intensity‐appropriate exercise in older adults with diabetes.

Smoking among adults with diabetes in Germany has remained relatively stable or shown only minor fluctuations over the last two decades [22, 48, 49]. Smoking decreases sharply with age, falling from 24%–25% among 40‐ to 59‐year‐olds to 11.4% among 70‐ to 79‐year‐olds, reaching 3.6% among those over 80 years [32]. Given the average age of 76 years in our study, it is not surprising that over 90% achieved the non‐smoking target, which remained stable in the age group of 66–79 years over time.

### 4.6 Strengths and Limitations

The major strength of this analysis lies in the presentation of nationwide diabetes care data for older and very old adults in Germany. Solid data was obtained through face‐to‐face medication interviews and examinations during home visits, which allowed access to those with disabilities or living in nursing homes. Our study has several limitations. First, we cannot exclude selection bias. Only about 40% of the Gesundheit65+ participants at baseline were able to complete the examination part. However, survey weights were used to account for losses during follow‐up, ensuring national representativeness. Second, data on diabetes type was not collected, that may have an impact on insulin use, but we assume that most study participants had T2D [3]. Third, this analysis included only selected indicators of diabetes care. Data on intermediate outcomes, such as blood glucose and cholesterol levels, and process indicators, such as medical eye and foot examinations, as well as diabetic‐specific comorbidities, such as chronic kidney disease, were not collected, preventing us from evaluating diabetes care more comprehensively. Finally, the number of cases in some subgroups is small, so the results should be interpreted with caution and with consideration of confidence intervals. Of note, due to the limited sample size of persons with diabetes and comorbid CVD in the age group 66–79 years, no cases of GLP‐1RA/SGLT2i use were reported in the 2008–2011 survey (Figure 2). This, however, does not reflect the actual clinical practice in Germany at the time, as the first GLP‐1RA was introduced in 2006.

## 5 Conclusions

Among older adults with diabetes in Germany, nearly three‐quarters are treated with antidiabetic medications, two‐thirds take statins, and almost 90% of those with hypertension receive ACEI/ARBs. Nevertheless, guideline‐based medication use is only partially optimal, with results suggesting substantial room for improvement for novel glucose‐lowering medications with cardiorenal benefits and aspirin use for secondary prevention of CVD. While most older adults with diabetes meet guideline‐based recommendations for non‐smoking, non‐obesity, and BP control, fewer than half engage in physical exercise. Thus, further improvements in health behaviours—especially physical exercise—and cardiometabolic risk factors need to be reinforced through behavioural and setting‐based interventions.

## Funding

This work was supported by Bundesministerium für Gesundheit (ZMVI1‐2518FSB410).

## Conflicts of Interest

The authors declare no conflicts of interest.

## Supporting information


**Table S1:** Proportion of older adults with diabetes achieving guideline‐based recommendations of diabetes care in the follow‐up in 2022/2023 of the Study on Health of Older People in Germany (Gesundheit65+, *N* = 292).
**Table S2:** Use of specific medications among older adults with diabetes in the follow‐up in 2022/2023 of the Study on Health of Older People in Germany (Gesundheit65+, *N* = 292).

## Data Availability

The data that support the findings of this study are available on request from the corresponding author. The data are not publicly available due to privacy or ethical restrictions.

## References

[1] A. Sinclair , P. Saeedi , A. Kaundal , S. Karuranga , B. Malanda , and R. Williams , “Diabetes and Global Ageing Among 65–99‐Year‐Old Adults: Findings From the International Diabetes Federation Diabetes Atlas, 9th Edition,” Diabetes Research and Clinical Practice 162 (2020): 108078. 10.1016/j.diabres.2020.108078 32068097

[2] Robert Koch Institute , “Diabetes Mellitus: Prevalence (From 18 Years),” (2025), www.gbe.rki.de/diabetes‐prevalence.

[3] L. Reitzle , P. Ihle , C. Heidemann , R. Paprott , I. Köster , and C. Schmidt , “Algorithm for the Classification of Type 1 and Type 2 Diabetes Mellitus for the Analysis of Routine Data,” Gesundheitswesen 85, no. S 02 (2023): S119–s126. 10.1055/a-1791-0918 35654399

[4] J. Baumert , L. Reitzle , R. Brinks , R. Kuhnert , and C. Heidemann , “Trends in Prevalence and Number of Cases of Diagnosed Type 2 Diabetes in Germany: Projections Until 2050,” Journal of Health Monitoring 10, no. 3 (2025): e13381. 10.25646/13381 PMC12478463 41030674

[5] J. Hill , “The Older Person With Diabetes: Considerations for Care,” British Journal of Community Nursing 24, no. 4 (2019): 160–164. 10.12968/bjcn.2019.24.4.160 30969848

[6] A. Milanesi and J. E. Weinreb , “Diabetes in the Elderly,” in Endotext, ed. K. R. Feingold , S. F. Ahmed , B. Anawalt , et al. (MDText.com, Inc, 2000).

[7] H. Calsbeek , N. A. B. M. Ketelaar , M. J. Faber , M. Wensing , and J. Braspenning , “Performance Measurements in Diabetes Care: The Complex Task of Selecting Quality Indicators,” International Journal for Quality in Health Care 25, no. 6 (2013): 704–709. 10.1093/intqhc/mzt073 24150483

[8] J. H. Hwang , M. K. Ali , and E. S. Huang , “Quality of Care in Adults With Diabetes,” in Diabetes in America 2023, ed. J. M. Lawrence , S. S. Casagrande , W. H. Herman , et al. (National Institute of Diabetes and Digestive and Kidney Diseases (NIDDK), 2023), https://www.ncbi.nlm.nih.gov/books/NBK612584/. 40063710

[9] M. L. McCreary , R. O. Yeung , D. P. Manca , M. Greiver , A. G. Singer , and D. Lau , “Use of Sodium‐Glucose Cotransporter‐2 Inhibitors in Adults Aged ≥ 65 Years With Type 2 Diabetes and Cardiovascular Disease: A Cross‐Sectional Study of Different Drug Funding Policies,” Canadian Journal of Diabetes 48, no. 8 (2024): 524–530.e522. 10.1016/j.jcjd.2024.09.003 39303780

[10] B. Safieddine , F. Trachte , S. Sperlich , J. Epping , K. Lange , and S. Geyer , “Trends of Antidiabetic and Cardiovascular Diseases Medication Prescriptions in Type 2 Diabetes Between 2005 and 2017‐A German Longitudinal Study Based on Claims Data,” International Journal of Environmental Research and Public Health 20, no. 5 (2023): 4491. 10.3390/ijerph20054491 PMC10001865 36901500

[11] N. Mardetko , U. Nabergoj Makovec , I. Locatelli , A. Janez , and M. Kos , “Uptake of New Antidiabetic Medicines in 11 European Countries,” BMC Endocrine Disorders 21, no. 1 (2021): 127. 10.1186/s12902-021-00798-3 PMC8235847 34172020

[12] K. Nong , B. T. Jeppesen , Q. Shi , et al., “Medications for Adults With Type 2 Diabetes: A Living Systematic Review and Network Meta‐Analysis,” BMJ 390 (2025): e083039. 10.1136/bmj-2024-083039 40813122

[13] A. Agarwal , R. Mustafa , V. Manja , et al., “Cardiovascular, Kidney Related, and Weight Loss Effects of Therapeutics for Type 2 Diabetes: A Living Clinical Practice Guideline,” BMJ 390 (2025): e082071. 10.1136/bmj-2024-082071 PMC12355350 40813129

[14] E. Marilly , J. Cottin , N. Cabrera , et al., “SGLT2 Inhibitors in Type 2 Diabetes: A Systematic Review and Meta‐Analysis of Cardiovascular Outcome Trials Balancing Their Risks and Benefits,” Diabetologia 65, no. 12 (2022): 2000–2010. 10.1007/s00125-022-05773-8 35925319

[15] D. Giugliano , M. Longo , S. Signoriello , et al., “The Effect of DPP‐4 Inhibitors, GLP‐1 Receptor Agonists and SGLT‐2 Inhibitors on Cardiorenal Outcomes: A Network Meta‐Analysis of 23 CVOTs,” Cardiovascular Diabetology 21, no. 1 (2022): 42. 10.1186/s12933-022-01474-z PMC8925229 35296336

[16] N. Marx , M. Federici , K. Schütt , et al., “2023 ESC Guidelines for the Management of Cardiovascular Disease in Patients With Diabetes,” European Heart Journal 44, no. 39 (2023): 4043–4140. 10.1093/eurheartj/ehad192 37622663

[17] American Diabetes Association Professional Practice Committee. 10. Cardiovascular Disease and Risk Management: Standards of Care in Diabetes—2025,” Diabetes Care 48 (2025): S207–S238. 10.2337/dc25-S010 PMC11635050 39651970

[18] Bundesärztekammer (BÄK), Kassenärztliche Bundesvereinigung (KBV), Arbeitsgemeinschaft der Wissenschaftlichen Medizinischen Fachgesellschaften (AWMF). “Nationale VersorgungsLeitlinie Typ‐2‐Diabetes—Langfassung, Version 3, 2023”, DIO: 10.6101/AZQ/000503, www.leitlinien.de/diabetes.

[19] American Diabetes Association Professional Practice Committee. 8. Obesity and Weight Management for the Prevention and Treatment of Type 2 Diabetes: Standards of Care in Diabetes–2025,” Diabetes Care 48, no. Supplement_1 (2025): S167–S180. 10.2337/dc25-S008 PMC11635032 39651976

[20] American Diabetes Association Professional Practice Committee. 5. Facilitating Positive Health Behaviors and Well‐Being to Improve Health Outcomes: Standards of Care in Diabetes—2025,” Diabetes Care 48, no. Supplement_1 (2025): S86–S127. 10.2337/dc25-S005 PMC11635047 39651983

[21] Bundesärztekammer (BÄK), Kassenärztliche Bundesvereinigung (KBV), Arbeitsgemeinschaft der Wissenschaftlichen Medizinischen Fachgesellschaften (AWMF) “Nationale VersorgungsLeitlinie Typ‐2‐Diabetes, Ergänzung zu Version 3: Kapitel Nicht‐Medikamentöse Therapie 2024”, DIO: 10.6101 AZQ 000518, https://register.awmf.org/de/leitlinien/detail/nvl‐001.

[22] Y. Du , C. Heidemann , A. Schaffrath Rosario , et al., “Changes in Diabetes Care Indicators: Findings From German National Health Interview and Examination Surveys 1997‐1999 and 2008‐2011,” BMJ Open Diabetes Research & Care 3, no. 1 (2015): e000135. 10.1136/bmjdrc-2015-000135 PMC4653864 26629347

[23] Y. Du , J. Baumert , R. Paprott , H. Neuhauser , C. Heidemann , and C. Scheidt‐Nave , “Gender Differences in Cardiovascular Risk Profiles and Diabetes Care Among Adults With Type 2 Diabetes in Germany,” Diabetes & Metabolism 45, no. 2 (2019): 204–206. 10.1016/j.diabet.2018.05.011 29954666

[24] W. Thefeld , H. Stolzenberg , and B. M. Bellach , “The Federal Health Survey: Response, Composition of Participants and Non‐Responder Analysis,” Gesundheitswesen 61 (1999): S57–S61. 10726397

[25] C. Scheidt‐Nave , P. Kamtsiuris , A. Gößwald , et al., “German Health Interview and Examination Survey for Adults (DEGS)—Design, Objectives and Implementation of the First Data Collection Wave,” BMC Public Health 12 (2012): 730. 10.1186/1471-2458-12-730 PMC3490742 22938722

[26] J. Fuchs , B. Gaertner , H. Perlitz , et al., “Study on Health of Older People in Germany (Gesundheit 65+): Objectives, Design and Implementation,” Journal of Health Monitoring 8, no. 3 (2023): 61–83. 10.25646/11666 PMC10565879 37829118

[27] National Diabetes Surveillance at the Robert Koch Institute. “Results of Diabetes Surveillance 2015‐2024. Type 2 diabetes medication—Adults (Robert Koch‐Institut, 2024).

[28] H. Brauns , S. Scherer , and S. Steinmann , “The CASMIN Educational Classification in International Comparative Research,” in Advances in Cross‐National Comparison: A European Working Book for Demographic and Socio‐Economic Variables, ed. J. H. P. Hoffmeyer‐Zlotnik and C. Wolf (Springer US, 2003), 221–244.

[29] C. Heidemann , Y. Du , E. Mauz , et al., “Healthcare and Health Situation of Adults With Type 2 Diabetes in Germany: The Study GEDA 2021/2022‐Diabetes,” Journal of Health Monitoring 9, no. 2 (2024): e12128. 10.25646/12128 PMC11262738 39081469

[30] T. Otto , J. Diesing , J. Borchert , et al., “Age‐Dependent Prevalence of Type 2 Diabetes, Cardiovascular Risk Profiles and Use of Diabetes Drugs in Germany Using Health Claims Data,” Diabetes, Obesity & Metabolism 25, no. 3 (2023): 767–775. 10.1111/dom.14924 36457132

[31] A. Zeyfang , S. Gölz , F. Iraci , et al., “Pattern of Antidiabetic Drug Prescription in Older Persons With Type 2 Diabetes: Results From the German DPV Registry,” Journal of the American Medical Directors Association 26, no. 8 (2025): 105724. 10.1016/j.jamda.2025.105724 40553729

[32] DMP Atlas Nordrhein‐Westfalen. “DMP Diabetes Mellitus Typ 2,” https://www.zi‐dmp.de/dmp‐atlas_nrw/index.html#diabetes‐mellitus‐typ‐2.

[33] B. Mertes , N. Zeise , S. Gödde , et al., “Insulin Prescribing in Accordance With the Guidelines for Type II Diabetes: An Analysis of Billing Data of a German Statutory Health Insurance Carrier, the Allgemeine Ortskrankenkasse (AOK) Hessen,” Deutsches Ärzteblatt International 123, no. 9 (2026): 235–239. 10.3238/arztebl.m2026.0009 PMC13288008 41668322

[34] American Diabetes Association Professional Practice Committee. 13. Older Adults: Standards of Care in Diabetes—2025,” Diabetes Care 48 (2025): S266–S282. 10.2337/dc25-S013 PMC11635042 39651977

[35] By the American Geriatrics Society Beers Criteria^(R)^ Update Expert Panel. American Geriatrics Society 2023 Updated AGS Beers Criteria^(R)^ for Potentially Inappropriate Medication Use in Older Adults,” Journal of the American Geriatrics Society 71, no. 7 (2023): 2052–2081. 10.1111/jgs.18372 PMC12478568 37139824

[36] K. Kostev , S. Gölz , B. M. Scholz , M. Kaiser , and S. Pscherer , “Time to Insulin Initiation in Type 2 Diabetes Patients in 2010/2011 and 2016/2017 in Germany,” Journal of Diabetes Science and Technology 13, no. 6 (2019): 1129–1134. 10.1177/1932296819835196 PMC6835192 30862186

[37] “Arzneimittel‐Atlas. Der Arzneimittelverbrauch in der GKV. Die wichtigsten Ergebnisse,” Medizinisch wissenschaftliche Verlagsgesellschaft, edited by B. Häussler and A. Höer (Hrsg.), (IGES Institut GmbH, 2025).

[38] G. A. Silverii , E. Caldini , I. Dicembrini , M. Pieri , M. Monami , and E. Mannucci , “Deprescription in Elderly Patients With Type 2 Diabetes Mellitus,” Diabetes Research and Clinical Practice 170 (2020): 108498. 10.1016/j.diabres.2020.108498 33068664

[39] American Diabetes Association Professional Practice Committee. 9. Pharmacologic Approaches to Glycemic Treatment: Standards of Care in Diabetes—2025,” Diabetes Care 48 (2025): S181–S206. 10.2337/dc25-S009 PMC11635045 39651989

[40] N. Hennies , S. W. Görgens , J. Killer , et al., “Prevalence of Obesity and Cardiovascular Disease in Adults With Type 2 Diabetes and Use of Diabetes Medication in Germany: A Claims Data Study,” Diabetes, Obesity & Metabolism 26, no. 12 (2024): 5636–5645. 10.1111/dom.15931 39300717

[41] S. Swamy , S. M. Noor , and R. O. Mathew , “Cardiovascular Disease in Diabetes and Chronic Kidney Disease,” Journal of Clinical Medicine 12, no. 22 (2023): 6984. 10.3390/jcm12226984 PMC10672715 38002599

[42] J. Kong , S. Park , T. H. Kim , et al., “Worldwide Burden of Antidiabetic Drug‐Induced Sarcopenia: An International Pharmacovigilance Study,” Archives of Gerontology and Geriatrics 129 (2025): 105656. 10.1016/j.archger.2024.105656 39447350

[43] M. Auzanneau , J. Seufert , S. Zimny , et al., “Regional Socioeconomic Deprivation Associated With the Use of Sodium‐Glucose Cotransporter‐2 Inhibitors and Glucagon‐Like Peptide‐1 Receptor Agonists in Adults With Type 2 Diabetes in Germany,” Diabetes, Obesity and Metabolism 26, no. 9 (2024): 4116–4120. 10.1111/dom.15741 38982585

[44] World Health Organization (WHO) , “First‐Ever Global Coverage Targets for Diabetes Adopted at the 75th World Health Assembly,” (2022), https://www.who.int/news‐room/feature‐stories/detail/first‐ever‐global‐coverage‐targets‐for‐diabetes‐adopted‐at‐the‐75‐th‐world‐health‐assembly.

[45] F. Mach , C. Baigent , A. L. Catapano , et al., “2019 ESC/EAS Guidelines for the Management of Dyslipidaemias: Lipid Modification to Reduce Cardiovascular Risk: The Task Force for the Management of Dyslipidaemias of the European Society of Cardiology (ESC) and European Atherosclerosis Society (EAS),” European Heart Journal 41, no. 1 (2019): 111–188. 10.1093/eurheartj/ehz455 31504418

[46] Y. Saito , S. Okada , H. Ogawa , et al., “Low‐Dose Aspirin for Primary Prevention of Cardiovascular Events in Patients With Type 2 Diabetes Mellitus,” Circulation 135, no. 7 (2017): 659–670. 10.1161/CIRCULATIONAHA.116.025760 27881565

[47] L. Bowman , M. Mafham , K. Wallendszus , et al., “Effects of n‐3 Fatty Acid Supplements in Diabetes Mellitus,” New England Journal of Medicine 379, no. 16 (2018): 1540–1550. 10.1056/NEJMoa1804989 30146932

[48] M. Laxy , G. Knoll , M. Schunk , C. Meisinger , C. Huth , and R. Holle , “Quality of Diabetes Care in Germany Improved From 2000 to 2007 to 2014, but Improvements Diminished Since 2007. Evidence From the Population‐Based KORA Studies,” PLoS One 11, no. 10 (2016): e0164704. 10.1371/journal.pone.0164704 PMC5066975 27749939

[49] M. Mehring , E. Donnachie , F. C. Bonke , C. Werner , and A. Schneider , “Disease Management Programs for Patients With Type 2 Diabetes Mellitus in Germany: A Longitudinal Population‐Based Descriptive Study,” Diabetology and Metabolic Syndrome 9, no. 1 (2017): 37. 10.1186/s13098-017-0236-y PMC5437566 28529546

[50] J. Becker , K. M. F. Emmert‐Fees , G. G. Greiner , et al., “Associations Between Self‐Management Behavior and Sociodemographic and Disease‐Related Characteristics in Elderly People With Type 2 Diabetes—New Results From the Population‐Based KORA Studies in Germany,” Primary Care Diabetes 14, no. 5 (2020): 508–514. 10.1016/j.pcd.2020.01.004 32088161

[51] A. Brown , N. Guess , A. Dornhorst , S. Taheri , and G. Frost , “Insulin‐Associated Weight Gain in Obese Type 2 Diabetes Mellitus Patients: What Can Be Done?,” Diabetes, Obesity & Metabolism 19, no. 12 (2017): 1655–1668. 10.1111/dom.13009 28509408

[52] S. Linder , K. Abu‐Omar , W. Geidl , et al., “Physical Inactivity in Healthy, Obese, and Diabetic Adults in Germany: An Analysis of Related Socio‐Demographic Variables,” PLoS One 16, no. 2 (2021): e0246634. 10.1371/journal.pone.0246634 PMC7872299 33561175

