# Supplementary material for: Quality of Diabetes Care Among Older Adults in Germany—Results of the Study on Health of Older People 2022/2023 Compared With Previous National Health Surveys
Source: Diabetes Obes Metab. 2026 Aug 11;28(11):10018–27. doi: 10.1111/dom.71184 (PMC13646352; doi:10.1111/dom.71184)
Supplement: Supplementary file 1 [file DOM-28-10018-s001.docx]

**Appendix:**

**Table S1:** **Proportion of older adults with diabetes achieving guideline-based recommendations of diabetes care in the follow-up in 2022/2023 of the Study on Health of Older People in Germany (Gesundheit65+*,* N=292)**

|  | **Men (n=182)** | | **Women (n=110)** | | p |
| --- | --- | --- | --- | --- | --- |
|  | % | 95% CI | % | 95% CI |  |
| **Self-management of health behavior** |  |  |  |  |  |
| Physical exercise | 39.2 | 28.2-50.2 | 51.5 | 39.5-63.6 | 0.149 |
| Non-smoking | 87.6 | 79.9-95.3 | 93.7 | 86.6-100 | 0.242 |
| **Cardiometabolic factor** |  |  |  |  |  |
| Non-obesity (BMI <30 kg/m^2^) | 73.8 | 63.0-84.6 | 64.1 | 52.4-75.8 | 0.190 |
| Blood pressure control  (SBP <140 & DBP <90) | 79.4 | 70.8-88.1 | 68.8 | 58.9-78.7 | 0.156 |
| **Medication use** |  |  |  |  |  |
| Statin use | 69.2 | 59.1-79.2 | 62.8 | 49.6-76.0 | 0.455 |
| ACEI/ARB in persons with hypertension | 91.8 | 85.7-97.9 | 84.8 | 76.1-93.6 | 0.248 |
| ACEI/ARB in persons with CVD | 79.7 | 67.3-92.0 | 90.3 | 80.8-99.9 | 0.206 |
| GLP-1RA/SGLT2i in persons with CVD | 36.8 | 20.6-53.0 | 37.3 | 21.8-52.8 | 0.962 |
| Aspirin in persons with CVD | 43.6 | 27.3-59.9 | 73.6 | 58.2-89.0 | 0.025 |

*CI: confidence interval. BMI: body mass index. SBP<140 & DBP <90: systolic blood pressure <140 mm Hg & diastolic blood pressure <90 mm Hg. ACEI/ARB: angiotensin-converting enzyme inhibitor/angiotensin receptor blocker. CVD: cardiovascular disease. GLP -1RA/SGLT2i: Glucagon-like peptide-1 receptor agonist/Sodium-glucose co-transporter 2 inhibitor.*

*P values: comparison between men and women within group. Weighted analyses, adjusted for age and educational level.*

**Table S2: Use of specific medications among older adults with diabetes in the follow-up in 2022/2023 of the Study on Health of Older People in Germany (Gesundheit65+*,* N=292)**

|  |  | **Total (N=292)** | | | **Men (n=182)** | | | **Women (n=110)** | | | **p** |
| --- | --- | --- | --- | --- | --- | --- | --- | --- | --- | --- | --- |
|  |  | n | % | 95% CI | n | % | 95% CI | n | % | 95% CI |  |
| **Antihyperglycemics** | any use | 223 | 73.6 | 65.7-80.2 | 155 | 84.7 | 75.4-90.9 | 68 | 61.1 | 47.0-73.6 | 0.006 |
|  | *- GLP-1RA* | 9 | 3.2 | 1.5-6.9 | 6 | 3.5 | 1.4-8.4 | 3 | 2.9 | 0.8-10.8 | 0.840 |
|  | *-* SGLT2i | 67 | 22.4 | 16.2-30.2 | 47 | 27.0 | 19.0-36.8 | 20 | 17.3 | 9.4-29.6 | 0.156 |
|  | *- GLP-1 RA and/or SGLT2i* | 72 | 23.5 | 17.2-31.3 | 51 | 28.8 | 20.8-38.5 | 21 | 17.5 | 9.6-29.8 | 0.099 |
|  | *- DPP-4i* | 64 | 24.6 | 17.9-32.8 | 45 | 28.7 | 21.2-37.5 | 19 | 20.0 | 11.4-32.6 | 0.166 |
| **Antihypertensives** | any use | 252 | 88.8 | 82.6-93.0 | 155 | 90.2 | 83.4-94.4 | 97 | 87.3 | 76.1-93.6 | 0.539 |
|  | *- ACEI/ARB* | 211 | 76.6 | 69.8-82.2 | 132 | 79.4 | 70.9-86.0 | 79 | 73.3 | 61.4-82.6 | 0.367 |
| **Antihyperlipidemics** | any use | 184 | 67.1 | 58.2-75.0 | 118 | 68.8 | 59.2-77.0 | 69 | 65.3 | 49.9-78.0 | 0.671 |
| **PAI** | any use | 119 | 38.7 | 31.0-47.1 | 73 | 43.5 | 33.8-53.7 | 46 | 33.3 | 22.5-46.2 |  |
|  | *- aspirin* | 110 | 35.1 | 27.8-43.2 | 67 | 39.7 | 30.5-49.7 | 43 | 30.0 | 19.9-42.5 | 0.209 |

*CI: confidence interval. GLP -1RAGlucagon-like peptide-1 receptor agonist. SGLT2i: Sodium-glucose co-transporter 2 inhibitor. DPP-4i: Dipeptidyl peptidase 4 inhibitor. PAI: platelet aggregation inhibitor, ACEI/ARB: angiotensin-converting enzyme inhibitor/angiotensin receptor blocker.*

*P values: comparison between men and women within group. Weighted analyses*
